# The Value of Creativity for Enhancing Translational Ecologies, Insights, and Discoveries

**DOI:** 10.3389/fpsyg.2019.00951

**Published:** 2019-07-09

**Authors:** Brian Goeltzenleuchter, Anna van Suchtelen, Kelly L. Brown, Gianfranco Grompone

**Affiliations:** ^1^Institute for Public and Urban Affairs, San Diego State University, San Diego, CA, United States; ^2^The Weber Honors College, San Diego State University, San Diego, CA, United States; ^3^Associate Faculty, Eureka Institute for Translational Medicine, Syracuse, Italy; ^4^Department of Pediatrics, Faculty of Medicine, The University of BC and BC Children's Hospital Research Institute, Vancouver, BC, Canada; ^5^Discovery Lead Nutrition and Health Science, Corporate R&D, Lesaffre International, Lille, France

**Keywords:** creativity, collaboration, translational research, uncertainty, divergent thinking, science education

## Introduction

The conventional scientific approach dictates use of higher-order analytical and systematic thinking to generate original solutions to problems. The act of originality however implies a departure from normality and the constraints of conventional boundaries. Creativity—the most complex and abstract higher order cognitive skill—likely lies at the core of innovative, solution-based thought by scientists (DeHann, 2011). Yet there is little if any formal teaching of creativity in the sciences, and “art” and “science” continue to mistakenly be portrayed on opposing ends of a spectrum of innate cognitive function.

As the research landscape continues to shift toward increasing complex interprofessional and multidisciplinary initiatives, scientists more than ever need to draw on creative thought processes not just to fuel discovery but to assemble and manage larger and more complicated collaborative relationships. It will be essential to incorporate scientific creativity in education and training programs for the next generation of scientists (DeHaan, 2009; Ness, 2011; Spoelstra et al., 2014; Foster and Lemus, 2015).

While applicable to all realms of science, in this article we use an example from translational medical research to illustrate the diversity of players that can be involved in scientific discovery. Consistent with the theme of originality, our article will take the unconventional form of a fable. Our aims are to (i) emphasize the importance of incorporating creativity into existing research programs, (ii) encourage readers to explore their own creative style, and (iii) provide a first step to educate trainees in the use of creative thought (see “Call to Action” insert) to enable convergent and divergent thinking within science's rule-bound system.

## Case Study: The Valley Crossing

How endlessly long the valley was. Far more hot and dry than our travelers could have imagined.

“Oh no!” Fox froze.

Horse bumped into Fox. “Excuse me,” he mumbled.

Parrot, who had been catching a ride on Horse's back, flapped his wings, and squawked. “Eek! A mouse! A dead mouse!”

Fox howled. Mouse looked up with fright. Goose One and Goose Two divided themselves: one at the front and one at the back of the group. Together they formed a protective wall.

“A dead mouse! A dead mouse!” This was Parrot again—who else—given to repeating everything he saw and heard.

The group was used to seeing skeletons. In fact, the trail was paved with them. But this was the first carcass they encountered on their journey, and it was startling. They had been plodding along for what seemed like an eternity. Dehydration, heat stroke, and exhaustion were exacerbated by insurmountable rocks, never-ending hills, and crisscrossing paths.

“What will it be this time? Left or right?” Horse asked jokingly. “Straight on! For sure!” Fox seethed.

“Squeak, squeak,” said Mouse but no one heard.

The truth was, they had no idea. They had lost their way long ago. With each step, each wrong turn, they were getting too tired to continue. But they had to keep going. They had come from afar, this brave group of six, and were determined to reach their destination on the other side of the valley. Time was running out though—they had to get there before they would be enveloped by the pitch dark and the freezing cold of night.

At home the wanderers were pillars in their community. But here, in this inhospitable environment, their skills were useless. All they could think to do was stay as optimistic as possible, but the stress was getting to them.

“I'm drenched in sweat,” said Horse, noting the irony of wading through the cracked dry mud of a riverbed that in wintertime would flood the valley with its wild and dangerous torrents. But now, in the middle of a long hot summer, there wasn't a drop of water in sight, apart from the beads of sweat that rolled from Horse's dusty coat. Like Horse, salty drops dripped into Fox's eyes. Horse, being a real workhorse, was used to this. But Fox wasn't. She could barely see her own paw. Both her vision and mind were becoming clouded. She craved a juicy chicken (she simply loved bird meat) and it took all her strength to not lick her lips around Goose One and Goose Two. She couldn't, they were partners after all and they had to work together. Furthermore: one doesn't (usually) eat colleagues.

As they walked on, the broken skeletons of mice became mere crackles beneath their feet. Suddenly, though, they stumbled over a skeleton that made them pause in horror.

“It's a goose!” Parrot shrieked. “Oh my! A dead goose! A dead goose!” Parrot's cries sounded more excited than horrified. “A dead goose!” Parrot couldn't stop himself.

Goose One and Goose Two refused to let themselves be disturbed. “Hold on,” they honked. “Hold on, we're good.” We won't end like this, they thought. Stay focused. “Straight on we go.”

But doubt had crept into the group miles ago.

“Squeak, squeak,” said Mouse but once again, no one listened.

“How can you be so sure?” Fox inquired of Goose One and Goose Two. “Didn't we get lost 100 times already?” “100 times! 200 times! 300!” Of course this was Parrot again.

“Oh come on, you. Get off my back,” Horse said to Parrot. He hated to waste time and just wanted to press on. Moreover, he was getting annoyed with Parrot's constant nattering.

“How can you two be so sure of this direction? I don't see any evidence here,” Fox persisted.

Goose One and Goose Two ignored them all and shrieked in a reassuring tone, “Hey guys, don't waste your energy, just follow us.” Though their air was full of self-confidence, the journey was getting to them too. Their neutral gray feathers were covered with a layer of filth that made them feel like grotesque crows. They craved a cool water bath in which they would clean their feathers with joyful splashing and playful hollering. With this in mind, they led their colleagues down a narrow path toward what looked to be a very promising vibrant green space with a luxurious pool. But they were fooled. There was no oasis.

“Wow! A mirage!” cheered Parrot. “A real mirage! Number one on my wish list!”

The other animals did not share Parrot's enthusiasm. Horse was especially fed up and aggressively shook his coat, flinging Parrot into the air. Mouse was increasingly weary; she had unsuccessfully been down this path before with other groups. They carried on, all six of them, despite their hardships, still focused on their collaborative mission.

Goose One was the first to see a bright white figure slowly moving in the distance. A mirage again? No. It actually looked like a living creature on its back. Sleeping? No. Napping? No. Sunbathing with a book! How could that be? The group was approaching a state of delirium. Sunburned and dehydrated as they were, no one wanted to admit what they saw. But the closer they got the more they believed, until all six froze in mid-step.

“QUACK!”

The whole group jumped, frightened by a high-pitched noise that came from the reclining figure.

“Well, my word!” said Fox, “It looks like a…”

“And it talks like a…” joined Horse.

“But then we still have to prove…” shrieked both geese in unison, though they were interrupted in mid sentence.

“Good afternoon!” said a ducky voice. “I'm Duck. Pleased to meet you. Don't be scared. It looks like you could use some help.”

A collective sigh of relief swept through the group as Duck inquired about the purpose of their journey. They all started talking at once. They just rattled away in a delirious state of exhaustion and excitement. “Help! Yes! That's what we need. We are done here! Do something! Anything!”

In the midst of the commotion, Duck waited patiently until the group was ready for her. She sat on a rock, wings folded behind her head, facing the burning sun. If she wouldn't have had a duck's beak, it would almost look like she was smiling. Her Zen-like ease calmed the group.

“Hey, come on, guys!” Goose Two said. “Let's stop for a moment.”

“Yeah,” Horse added, “Why not listen to what this duck has to say.”

“Might be bloody helpful,” said Parrot, actually expressing an original thought.

“But wait! This is a *duck*!” said Fox, while Duck calmly cleaned her white feathers with her beak. “Doesn't a duck need water?”

“Yeah, I would say so too,” neighed Horse. Goose One and Goose Two agreed.

Duck, who brought light to the group and not only because her feathers shone like the brightest sunbeams, stood up and started to walk. “Come on guys.” she said, “Let's walk and talk. And with her clumsy webbed feet she started to march so firmly and sure of herself that the group couldn't help but follow in awe. “I'm Duck,” Duck said, “and that's a good thing. I love a good problem. What's up?”

“Well listen,” they said. “We have a goal that's out of sight.”

“Then focus on me and not the goal,” Duck said.

From this moment on, Duck took the lead. The others followed her, eyes fixed on her white feathers and orange webbed feet, and ears tuned to her alarm-like quacks, which didn't seem so alarming anymore. During their walk, Duck did most of the talking. She explained to the group how important it was to reach the valley's end before dark, how they would turn into frozen statues if they didn't make it. But she didn't frighten the group with this rather unpleasant perspective. On the contrary: she taught her new acquaintances special survival tools.

“First of all,” Duck explained: “you might think you're a team, but you're not acting like one.” The group was too tired to argue, apart from some vague grumbling. “Let's work on your collective skills.”

Duck settled on the parched ground, convincing the pack to join her. Leaning against a formation of rocks, the group circled around her. Duck took stock of each member, pointing her beak at the animals, one at a time. First to both geese, whose protecting attitude had become too rigid during their travels. Then to Fox, who seemed to have lost his cunning entirely. Horse himself pointed out that his hard work didn't always amount to much. And Parrot, he was admonished for his constant jibber-jabbering. Lastly, Duck scolded the whole lot for not listening to Mouse.

“Squeak, squeak!” said Mouse, and suddenly the others heard “Speak, hear me speak!” And this time, for the very first time since entering the valley, they listened.

“Look at it this way,” Duck said reassuringly. “Parrot is a talker, so put him in a position to share his observations with the group. But Parrot, you need to use your wings to offer those observations. Fox, be shrewd about how you use your brute strength to overcome obstacles. Recognize when to use your nose, paws and teeth. Goose One and Two, use your eyes like I do.”

Yes, Duck mentioned the geese's eyes for a reason, as it pointed to her own strength: her vision. Duck's eyes were located at either side of her head, and capable of a 340-degree field of vision. She had a clear view of possible solutions, both nearby and on the periphery.

After some more exposition followed by a brainstorming session on how to use the desert to its full potential, they continued on their way. The valley, dry and hot as it was, happened to be Duck's habitat, which meant there was nothing to fear. Duck's way of guiding the group through the hostile landscape was quite extraordinary. What seemed like harsh terrain for the group was a playground for her, in which obstacles were toys. Duck showed the group that they could use barren trees as monkey bars, hilly paths as slides and sloped rocks as launch pads. Before long, the group joined Duck in whimsically reimagining their environment. They too started to jump, clamber, slide, and play with their surroundings. Parrot flew in front, narrating his birds-eye-view of upcoming hazards. Fox shrewdly dug holes around obstacles, set her teeth in roots in order to clear the path and create escape routes. Goose One and Goose Two took turns sharing their lead role with the others. Suddenly they found themselves more at ease in the middle, proudly helping Duck scan the desert with their bird's eyes, while making it a point to converse with Mouse about their direction. And Horse couldn't stop himself from being a workhorse: he carried each and every animal that needed a short rest on his back. That is, apart from Parrot, who was already sufficiently relaxed, and so pleased with his new role up in the air that he didn't even think of descending onto Horse's back anymore.

In the end, all hazards were warded off with the help of this lowland inhabitant who was so naturally at ease here in the arid valley. As the crimson-gold sky dissolved into the cold blue of evening, the group arrived at the valley's edge. They were bursting with curiosity, and now, with their goal in sight, they simply had to know.

“Duck, what about you? How can you possibly live here? How come you survive? How can you thrive here, in this valley of death?”

“It's your valley of death, not mine,” said Duck, while nodding to the horizon. High on a hilltop, a pasture green stretched out in front of Goose One and Goose Two, Fox, Horse, Mouse and Parrot, all ready to climb up. “I see it differently. I treat it differently. Like water off a duck's back.” Thus, spoke Duck, whose beak appeared, once again, to be smiling.

## Discussion

Through this fable we use animal symbolism to portray different stakeholders that may be present when crossing the so-called valley of death; this is an allegory for one of the many difficult paths of translating scientific discoveries into medicine practice reforms that in this fable is represented by the harsh landscape and physical barriers (Guilford, 1959; Gardner, 1983; Csikszentmihalyi, 1996; Robinson, 2009). The character of Duck is an applied metaphor for creativity that, when applied, unites and helps the group of basic scientists (Goose One and Goose Two), physicians (Horse), attorneys (Fox), journalists (Parrot), and patients (Mouse) overcome obstacles and reach their goal. We recognize that the characters and backdrop may differ depending on the actual nature of the stakeholders and goal. Yet regardless of context, different creative processes illustrated throughout the fable can augment the traditional knowledge and skills of translational science stakeholders:

### Divergent Thinking

In contrast to classical convergent thinking, which is primarily concerned with solving well-defined problems in order to arrive at the best, right, or conventional answer, a person uses divergent thinking to move from one known idea to imagining many possible solutions to a problem. The fluent, flexible and original spontaneous, non-linear manner in which divergent thinking is employed (Csikszentmihalyi, 1996) is exemplified in the fable through Duck's (and later, the Geese's) unique field of vision and the way it affected her understanding of the landscape (Guilford, 1959).

### Multiple Intelligences

Individuals have multiple forms of intelligence but often utilize only one or a few [dominant form(s)]; other forms are often dormant as opposed to being non-existent or weak (Gardner, 1983; Robinson, 2009). In our fable, the characters hold tight to skills they utilize in their day-to-day profession, failing to see the ineffectiveness of those skills in the desert, for e.g., Parrot's talkative nature and Fox's legal intelligence, while essential for their respective professions, are virtually useless in the valley (Butler, 2008). Eventually, Fox and Parrot collaborate and draw on innate skills (flying and digging) that were previously dormant in order to find nourishment for the group that in this context is representative of the process of findings investors for financial sustainability of research projects.

### Play and Flow

By far the easiest way into creative problem solving is to organize a team in which diverse skill sets are encouraged to freely collaborate and explore the sorts of unbiased and abstract ideas needed to solve “wicked” problems, and in which the experience is so enjoyable for all participants that they continue to do it even at great cost (Csikszentmihalyi, 2008). If the challenge the group is attempting to solve completely engages its collective skill set, the group will often find itself in a “flow” state, which in turn creates new skills to tackle more difficult challenges. In the fable, Duck reminds the group of their common purpose (Csikszentmihalyi, 1997; Butler, 2008; Fernandez-Moure, 2016) and eventually we see the animals losing themselves in the playground they once saw as a hostile environment, and taking turns sharing the load (Horse carrying different characters) and the lead. Leadership is negotiated, as when Goose One and Goose Two learned *when* to resist the temptation to venture down every fork in the road that, while appealing to their scientific curiosity and not technically a “wrong turn,” was exhaustive for the other team members.

We encourage the reader to consider and practice some of the mentioned creative processes; deliberate use of creativity in science communication (for example through the creation of a fable) is a laudable first step for scientists wishing to incorporate or enhance creativity in their program of research (see Box 1). In doing so, it may expedite scientific discovery, but most importantly, cognizant creative practice should allow scientists and non-scientists to maximize enjoyment that comes from collaborative discovery.

Box 1 **Call to action.**

An exercise to introduce creativity in the sciences. The intention of this fable is to provoke discussion among scientists regarding the role of creativity in scientific endeavor and science education. We encourage readers to use the fable as a focal point for such discussions and/or an example for trainees to craft their own creative work to explain complex scientific concepts.

## Author Contributions

All authors listed have made a substantial, direct and intellectual contribution to the work, and approved it for publication.

### Conflict of Interest Statement

The authors started to work on the manuscript in December 2017. GG is employed by Lesaffre International since May 2018. Prior to that he was employed by Instituto Nacional de Investigacion Agropecuaria, Uruguay, an academic institution. The remaining authors declare that this opinion article didn't receive any funding from Lesaffre International and that the research was conducted in the absence of any commercial or financial relationships that could be construed as a potential conflict of interest.
